# Concurrent Presentation of Diabetic Nephropathy and Type 1 Diabetes Mellitus in a Pediatric Patient

**DOI:** 10.7759/cureus.20831

**Published:** 2021-12-30

**Authors:** Sana Manazir, Hafiza M Durrani, Fatima Jawed, Hira Iqbal Naviwala

**Affiliations:** 1 Medicine and Surgery, Dow University of Health Sciences, Civil Hospital Karachi, Karachi, PAK; 2 Pediatrics, Dow University of Health Sciences, Civil Hospital Karachi, Karachi, PAK; 3 Internal Medicine, Dow University of Health Sciences, Civil Hospital Karachi, Karachi, PAK

**Keywords:** diabetic nephropathy (dn), type 1 diabetes mellitus (t1dm), endocrinology and diabetes, glycated haemoglobin (hba1c), pediatrics

## Abstract

Diabetic nephropathy (DN) is the most consequential and longstanding microvascular complication of type 1 diabetes mellitus (T1DM) and the most common cause for renal replacement therapy throughout the world. The most important risk factor for DN includes poor glycemic control. We present a rare case where biopsy-proven grade 3 DN had a concurrent presentation at the time of diagnosis of T1DM in a 12-year-old female child. This earlier than expected DN noted in this patient raises the question regarding the need for earlier surveillance for DN in children.

## Introduction

Diabetic nephropathy (DN) is a pernicious microvascular complication of diabetes mellitus (DM), and it is the foremost cause of end-stage renal disease (ESRD) in the West [1]. It has been stated that 25%-40% of type 1 diabetes mellitus (T1DM) patients and 5%-40% of type 2 DM (T2DM) patients will develop this deadly complication, thus making DN the leading cause for renal replacement therapy throughout the world [1-2].

We encountered a unique case of early DN in an adolescent, where initial presentation of T1DM was associated with biopsy-proven grade 3 DN. This case demonstrates that this microvascular complication of DM may also develop early and unexpectedly following the course of T1DM, and following routine screening guidelines for DN can lead to a possible delay in diagnosis as well as a potential increase in morbidity and mortality for the patients.

## Case presentation

A 12-year-old female child presented with polyuria, polydipsia and burning micturition for more than two months, along with joint pain and restriction of joint movements for two months, with associated mild shortness of breath on exertion (New York Heart Association Class I). There was no history of photosensitivity, oral ulcers, rash, alopecia, sore throat, chorea, chest pain and muscle weakness. Family history was positive for DM in maternal grandparents and hypertension in paternal grandmother. On examination, the child looked pale, well oriented, weighing 21 kg, with following vitals: afebrile, maintaining O_2_ saturation at room air, respiratory rate of 18/minute, heart rate of 130/minute, blood pressure of 115/70 mm Hg, and random blood sugar 445 mg/dl. On systemic examination, neurological examination, ophthalmological examination, cardiovascular examination and respiratory examination were unremarkable. Abdomen was soft, non-tender, hepatomegaly was appreciated 4 cm below the right coastal margin with firm consistency and smooth margins, and spleen was palpable 3 cm below the left coastal margin. Musculoskeletal examination showed mild tenderness and limited range of motion at bilateral ankle, knee, wrist and metacarpophalangeal joints; scleroderma was also present.

On investigation, complete blood count, serum urea, creatinine and electrolytes and liver function tests were unremarkable. The serum total protein level was 6.8 g/dl, serum albumin level was 2.2 g/dl and serum globulin level was 4.1 g/dl, with a protein albumin/globulin (A/G) ratio of 0.66. Lipid profile showed a cholesterol level of 267 mg/dl, triglyceride level of 581 mg/dl, high density lipoprotein level of 27 mg/dl and low density lipoprotein 124 mg/dl. Fasting blood sugar was 362 mg/dl and glycated hemoglobin, i.e. HbA1C, was >14.0%. Her random C-peptide (rCP) level was found to be low (<0.2 nmol/l) [3]. Thus, she was diagnosed as a case of T1DM, and subcutaneous insulin was started. Arterial blood gases were within the normal range. The detailed urine report of days 0, 1 and 6 is shown in Table 1.

**Table 1:** Detailed urine report of days 0, 1 and 6 of hospital admission HPF: high-power field

	Day 0	Day 1	Day 6
Color	Yellow	Yellow	Yellow
pH	5.5	5.5	8.0
Specific gravity	1.015	1.015	1.005
Protein	+++	+++	+++
Glucose	+++	+++	+++
Blood	+	Trace	Trace
Ketone bodies	+++	Nil	Nil
Nitrites	Nil	Nil	Nil
Red blood cells	8-10/HPF	4-6/HPF	6-8/HPF
Pus cells	2-3/HPF	1-2/HPF	4-6/HPF
Granular casts	++/HPF	+/HPF	Nil/HPF

**Figure 1:**
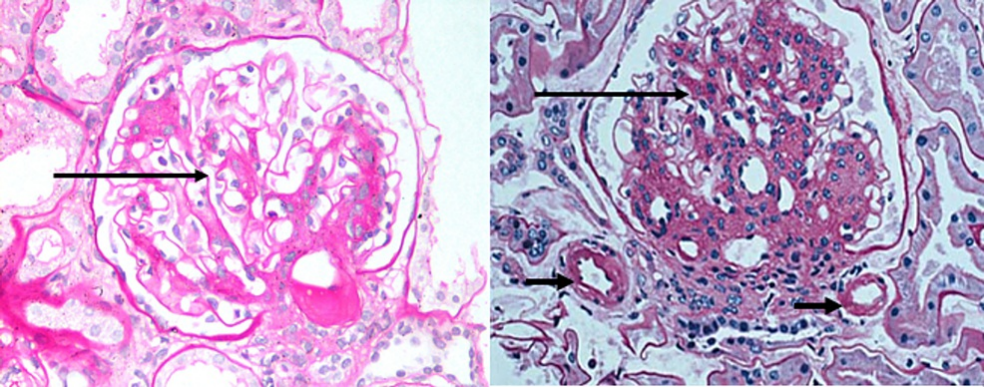
Diffuse and nodular mesangial expansion (long thin arrows) and afferent and efferent arteriolar hyalinosis (short thick arrows) on two glomeruli (periodic acid-Schiff stain)

**Figure 2:**
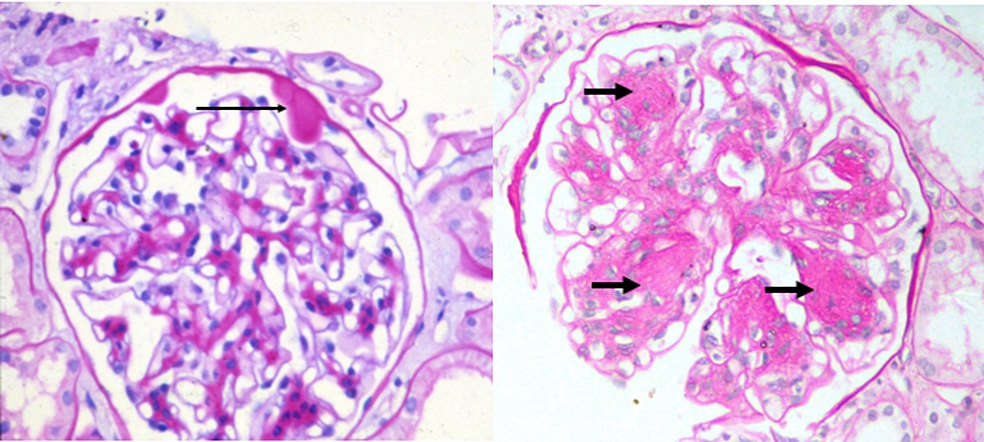
Left: A capsular drop lesion (long thin arrow). Right: glomeruli with nodular (Kimmelstiel-Wilson) lesions (short thick arrows) (periodic acid-Schiff stain)

Urine culture had growth of methicillin-resistant *Staphylococcus aureus* and *Klebsiella pneumoniae*, which was treated according to antibiotic sensitivities. Serum thyroid stimulating hormone was insignificant (0.68 µIU/ml). Electrocardiogram (ECG) and echocardiogram showed no abnormality. The immunological workup, which included anti-nuclear antibodies, anti-dsDNA (double stranded), rheumatoid arthritis factor, and anti-cyclic citrullinated peptide, was negative. A percutaneous renal biopsy demonstrated up to 16 glomeruli; of these, one was sclerosed, rest all showed diffuse mesangiocapillary pattern and thickening of the glomerular basement membrane. Foci of afferent and efferent arteriolar hyalinosis were seen (Figure 1). Mild patchy tubular atrophy was also seen. In addition, six glomeruli showed Kimmelstiel Wilson nodules, one showed fibrin cap and foam cells and one showed capsular drop lesion as shown in Figure 2. Immunofluorescence was done, which showed a completely negative panel. Thus, renal biopsy was concluded as grade 3 diabetic nephropathy.

## Discussion

DN is the most consequential and longstanding microvascular complication of T1DM; hyperglycemia induces reversible homeostatic abnormalities in blood flow and vascular permeability of renal glomeruli initially that eventually leads to irreversible damage [4]. Risk factors for the advancement of DN include poor glycemic control, renovascular hypertension, proteinuria, hyperlipidemia and genetic disposition, with HbA1C being an independent risk factor for cardiovascular morbidity in patients of T1DM [2]. In a population-based cohort study, conducted among children and adults diagnosed with T1DM, it was concluded that HbA1c levels of 7.0%-7.4% are associated with an increased risk of any retinopathy and microalbuminuria, whereas HbA1c levels of >8.6% pose an increased risk of proliferative retinopathy and macroalbuminuria [5]. More than one-third of T1DM and T2DM patients develop this microvascular complication, with the peak incidence at 10-20 years of disease onset; ethnicities such as African Americans, Asians, and Native Americans are more prone to acquire DN; this may progress to a more severe form of DN [1].

A study on a U.S. pediatric population stated that the incidence of DN has escalated from 1.16% to 3.44% between 2002 and 2013; the maximum prevalence was noted from the ages of 12 to less than 18 years, and female diabetics contributed to more cases of DN than males [6]. According to the literature, 45% of T1DM and T2DM patients will develop this deadly microvascular complication with the peak incidence after 10-20 years of disease onset [1]. Recently, a study conducted among patients of T1DM in Sudan reported a significantly high prevalence of microvascular chronic complications at a young age; the youngest patient with microalbuminuria aged 11 years and had diabetes duration of only three years [7]. In the initial stages of DN, microalbuminuria occurs that has been found as early as within the first two years after disease onset; later, macroalbuminuria and blatant nephropathy manifest, usually after 10 to 15 years of T1DM onset [2]. The latest goal of DN management includes a multifactorial approach and mainly aims strict glycemic control, blood pressure control and lipid control [1]. If measures are not taken promptly or timely, microalbuminuria may progress to macroalbuminuria and then to overt nephropathy and finally ESRD, which is the foremost cause of morbidity and mortality in T1DM patients.

## Conclusions

The prevalence of T1DM and DN in the pediatric population has increased in past years, with microalbuminuria being reportedly present during first three years of disease onset. Here, we presented a rare and unique case of a 12-year-old female child where the initial presentation of T1DM was associated with biopsy-proven grade 3 DN. Thus, we recommend the need for early surveillance of DN in children and adolescents with T1DM; also, large population studies are also recommended to know the actual prevalence of overt nephropathy at the time of diagnosis of T1DM, and further studies to focus on risk factors responsible for such precipitous nephropathy.
